# Differential regulation of TRP channel gene and protein expression by intervertebral disc degeneration and back pain

**DOI:** 10.1038/s41598-019-55212-9

**Published:** 2019-12-11

**Authors:** A. Sadowska, W. Hitzl, A. Karol, P. Jaszczuk, H. Cherif, L. Haglund, O. N. Hausmann, K. Wuertz-Kozak

**Affiliations:** 1 Institute for Biomechanics, D-HEST, ETH Zurich, Zurich, Switzerland; 2 Research Office - Biostatistics, Paracelsus Medical University, Salzburg, Austria; 3 Department of Ophthalmology and Optometry, Paracelsus Medical University, Salzburg, Austria; 4 Research Program Experimental Ophthalmology and Glaucoma Research, Paracelsus Medical University Salzburg, Salzburg, Austria; 5 Musculoskeletal Research Unit (MSRU), Department of Molecular Mechanisms of Disease (DMMD), Vetsuisse Faculty, University of Zurich, Zurich, Switzerland; 6 Klinik St. Anna-Hirslanden, Neuro- and Spine Center, Lucerne, Switzerland; 7 Orthopaedic Research Laboratory, Montreal, QC Canada; 8 McGill Scoliosis and Spine Research Group, Montreal, QC Canada; 9 The Shriners Hospital for Children, Montreal, QC Canada; 10 Department of Biomedical Engineering, Rochester Institute of Technology (RIT), Rochester, USA; 11 Spine Center, Schön Clinic Munich Harlaching, Academic Teaching Hospital and Spine Research Institute of the Paracelsus Medical University Salzburg, Germany

**Keywords:** Cell biology, Diseases, Chronic pain

## Abstract

Intervertebral disc (IVD) degeneration and consequent low back pain (LBP) are common and costly pathological processes that require improved treatment strategies. Transient Receptor Potential (TRP) channels constitute a family of multimodal ion channels that have recently emerged as contributors to disc pathologies and were thus proposed as potential therapeutic targets, although limited data on their presence and function in the IVD exist. The purpose of this study was to determine the mRNA and protein expression of TRP channels in non-degenerated and degenerated human IVD tissue (with different pain intensity and chronicity) using gene array, conventional qPCR and immunohistochemistry. We could demonstrate that 26 out of 28 currently known TRP channels are expressed in the IVD on the mRNA level, thereby revealing novel therapeutic candidates from the TRPC, TRPM and TRPML subfamilies. TRPC6, TRPM2 and TRPML1 displayed enhanced gene and protein expression in degenerated IVDs as compared to non-degenerated IVDs. Additionally, the gene expression of TRPC6 and TRPML1 was influenced by the IVD degeneration grade. Pain intensity and/or chronicity influenced the gene and/or protein expression of TRPC6, TRPM2 and TRML1. Interestingly, decreased gene expression of TRPM2 was observed in patients treated with steroids. This study supports the importance of TRP channels in IVD homeostasis and pathology and their possible application as pharmacological targets for the treatment of IVD degeneration and LBP. However, the exact function and activation of the highlighted TRP channels will have to be determined in future studies.

## Introduction

The intervertebral disc (IVD) is a mechanosensitive tissue that lies between adjacent vertebrae in the spinal column. The mechanical properties of the IVD are greatly defined by its biochemical composition, with the highly hydrated nucleus pulposus (NP) in the center, surrounded by the annulus fibrous (AF)^1^. The primary function of the IVD is to transmit loads arising from muscle activity and body weight, with hydrostatic pressure/compression and osmotic stresses predominating in the NP and tensile/shear stresses in the AF^1^. With its low cellularity (5000 cells/mm^3^ in the NP^2^), avascular structure (with a consequent lack of nutrients and oxygen), high daily mechanical loads and an inability to repair itself, the IVD is prone to early degeneration. Degeneration is associated with a loss in extracellular matrix (ECM) components, specifically proteoglycans, resulting in a consequent loss of tissue hydration as well as tissue weakening, including clefts and tears^3^. These changes not only influence the mechanical properties of the IVD, but also lead to high stress zones and hence altered IVD mechanobiology.

In a subgroup of those affected by IVD degeneration, inflammatory processes take place within the IVD tissue. Inflammation has been described as a major contributor to the development of painful disc degeneration (also known as degenerative disc disease (DDD)), hence presumably distinguishing symptomatic from asymptomatic IVD degeneration^4^. On the molecular level, DDD can be characterized by an up-regulation of pro-inflammatory cytokines such as interleukin (IL)-6, IL-1B and tumor necrosis factor (TNF)-α^3^. Moreover, mechanical loading can also induce inflammation, depending on its type, applied magnitude, duration and frequency^3,5,6^. Similarly, altered IVDs osmolarity can contribute to tissue inflammation by modulating pro-inflammatory mediators and pathways (e.g. mitogen-activated protein (MAP) kinases, T-cells 5/tonicity response element-binding protein (NFAT5/TonEBP))^7,8^ and molecules (e.g. IL-6, IL-1B)^9^.

Although inflammation as well as mechanical and osmotic stress have been identified as important factors in the development of painful disc degeneration and hence back pain, the exact pathobiological mechanism remain to this day unknown. However, a superfamily of multimodal ion channels, the so-called transient receptor potential (TRP) channels, have recently emerged as potential contributors to disc pathologies^10^. TRP channels are of utmost interest in IVD research as they are regulated by a diverse range of stimuli, including mechanical and osmotic stress, and furthermore modulate inflammatory responses and mediate a variety of sensations, including pain. Stimulation of a TRP channel will cause its activation (i.e. opens channels pore), leading to ion movement and resulting in elevated cytosolic intracellular calcium. Applied stimuli (e.g. shear stress) can change a channel’s molecular distribution, hence altering its membrane trafficking and spatial/temporal distribution, which in turn can influence its activity threshold levels^11,12^. Very recent studies highlighted TRPC6 and TRPV4 as possible contributors to the IVD’s health and disease^9,13–15^, but numerous other members of the various TRP families (ankyrin TRPA, canonical TRPC, vanilloid TRPV, melastatin TRPM, mucolipin TRPML and polycystic TRPP) have not yet been investigated.

To gain a better insight into the role of TRP channels in the IVD and low back pain, the purpose of this study was to identify the presence of all currently known TRP channels in non-degenerated and degenerated human IVDs with varying pain intensity and chronicity, and to highlight their possible involvement in IVD pathologies.

## Results

### TRP channel mRNA expression: Gene array

In the initial step, a wide screening of all 28 TRP channels was investigated in 8 IVD samples (4 degenerated: 2x NP and 2x AF, assessed with Pfirrmann classification^16^; and 4 non-degenerated: 2x NP and 2x AF, assessed with Thompson classification^17^) using a gene array. Out of 28 tested TRP channels, two targets (TRPC5 and TRPM5), were not detectable in any of the samples included in the gene array. For patient information, see Tables 1 and 2.

**Table 1:** Patient information.

Sample No.	Condition	Spine	Level	Region	Age	Sex	Pathology^#^	Degeneration grade*	Modic type
1	0	Lumbar	L1/L2	NP	51	m		2	n/a
2	0	Lumbar	L1/L2	AF	51	m		2	n/a
3	0	Lumbar	L2/L3	AF	53	f		2	n/a
4	0	Lumbar	L2/L4	NP	53	f		2	n/a
5	0	Lumbar	L3/L4	NP	27	m		2	n/a
6	0	Lumbar	L3/L4	AF	27	m		2	n/a
7	0	Thoracic and lumbar	L1/2-L2/3-L3/4	NP	34	m		2	n/a
8	0	Thoracic and lumbar	L1/2-L2/3-L3/4	AF	34	m		2	n/a
9	0	Lumbar	L1/2	NP	55	f		2	n/a
10	0	Lumbar	L1/2	AF	55	f		2	n/a
11	0	Lumbar	L1-L5, all four	NP	52	m		2	n/a
12	0	Thoracic and lumbar	T12-S1, all six	NP	17	m		2	n/a
13	1	Lumbar	L5/S1	NP	70	m	DH	4	1
14	1	Lumbar	L3/4	AF	80	m	DDD	4	2
15	1	Lumbar	L5/S1	AF	62	f	DDD	5	0
16	1	Lumbar	L5/S1	NP	59	f	DDD	5	0
17	1	Lumbar	L4/5	AF	66	f	DH	2	2
18	1	Lumbar	L5/S1	AF	33	m	DH	2	1
19	1	Lumbar	L5/S1	AF	39	m	DH	3	1
20	1	Cervical	C5/6	AF	47	f	DDD	3	1
21	1	Lumbar	L4/5 L5/S1	AF	31	m	DDD	4	2
22	1	Lumbar	L5/S1	AF	59	f	DDD	5	0
23	1	Lumbar	L5/S1	AF	46	f	DH	3	1
24	1	Lumbar	L5/S1	NP	54	f	DH	2	1
25	1	Lumbar	L5/S1	NP	46	f	DH	3	1
26	1	Lumbar	L4/5	NP	36	m	DH	3	2
27	1	Lumbar	L5/S1	NP	53	m	DH	3	1
28	1	Cervical	C6/7	NP	58	m	DDD	2	1
29	1	Lumbar	L4/5	NP	74	m	DH	4	2
30	1	Lumbar	L4/5	mix	55	m	DDD	3	2
31	1	Lumbar	L5/S1	AF	55	f	DDD	4	2
32	1	Lumbar	L5/S1	NP	50	f	DH	3	2
33	1	Cervical	C6/7	mix	78	f	DDD	4	2
34	1	Lumbar	L5/S1	NP	33	m	DDD	3	1

1 = degenerated; 0 = non-degenerated; NP = nucleus pulposus; AF = annulus fibrosus; m = male; f = female; DH = disc herniation; DDD = degenerative disc disease; n/a = data not available; # = not applicable to the healthy donors 1 to 12; * cadaveric samples were assessed with Thompson classification and degenerated biopsies with Pfirrmann classification.

**Table 2:** Patient information.

Sample No.	BMI	Steroids^#^	Smoking^#^	Pain score^#^	Duration of symptoms (grouped) ^#^	Cause of death*
1	24.39					n/a
2	24.39					n/a
3	20.49					Anoxia Carbon Monoxide
4	20.49					Anoxia Carbon Monoxide
5	n/a					Trauma
6	n/a					Trauma
7	22.72					Stroke
8	22.72					Stroke
9	n/a					Anoxia
10	n/a					Anoxia
11	n/a					Stroke
12	24.39					Brain death
13	24	1	0	3	2	
14	26	0	1	2	3	
15	25	0	0	1	3	
16	25	0	1	1	3	
17	25	1	0	2	2	
18	28	1	1	2	2	
19	27	0	0	2	3	
20	24	0	0	3	2	
21	26	1	1	2	3	
22	25	0	1	1	3	
23	18	1	1	1	3	
24	24	0	0	2	1	
25	18	1	1	1	3	
26	40	0	1	2	2	
27	27	1	1	1	2	
28	26	0	1	2	2	
29	23	0	0	2	3	
30	27	1	1	3	3	
31	23	1	0	2	3	
32	20	1	1	3	1	
33	21	1	0	3	1	
34	23	1	0	2	3	

Steroids or smoking: 0 = no, 1 = yes; Pain score: 1 = moderate pain, 2 = intense pain, 3 = disabling pain; Pain duration: 1: 1–2 months, 2: 2–12 months, 3: > 1 year; n/a = data not available; * = applicable only to the healthy donors one to 12; # = not applicable to cadaveric samples 1 to 12.

In the non-degenerated IVDs, TRPC1, TRPC2, TRPM7, TRPML1–3, PKD1, TRPP1 (PKD2), TRPV1, TRPV3 and TRPV4 were detected in both, NP and AF samples, whereas other TRP channels were only detectable in either region: TRPC6 (AF), TRPM4 (NP), TRPM6 (NP), and TRPV5 (NP). Furthermore, TRPA1, TRPC3, TRPC4, TRPC7, TRPM1-M3, TRPM8, TRPP2 (PKD2L1), TRPP3 (PKD2L2), TRPV2 and TRPV6 were undetectable in the selected non-degenerated IVD samples.

In the degenerated IVDs, the highest gene expression, detected in both NP and AF, was measured for TRPC1, TRPM7, PKD1, TRPP1 (PKD2) and TRPV4. TRPC7 was detected only in the AF (2 out of 2 degenerated AF samples) and TRPM1 only in the NP (2 out of 2 degenerated NP samples). TRPP3 (PKD2L2) could be detected only in one out of two AF sample.

**Figure 1:**
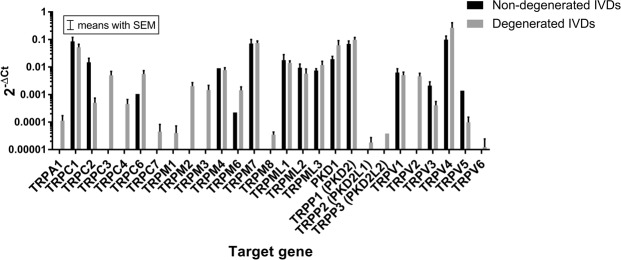
The overall TRP channels gene expression as detected during the array screening in the non-degenerated (n = 4, black bars) and degenerated (n = 4, grey bars) human IVD tissue. Data are presented on a logarithmic scale as 2^−ΔCt^ values (relative to YWHAZ).

Figure 1 presents overall (combined NP and AF) gene expression as measured on the gene array normalized to YWHAZ (measured Ct values for all tested TRP targets in the gene array screening are presented in the Supplementary Material Table S1).

### TRP channel expression (mRNA): Disease state and degeneration grade

**Figure 2:**
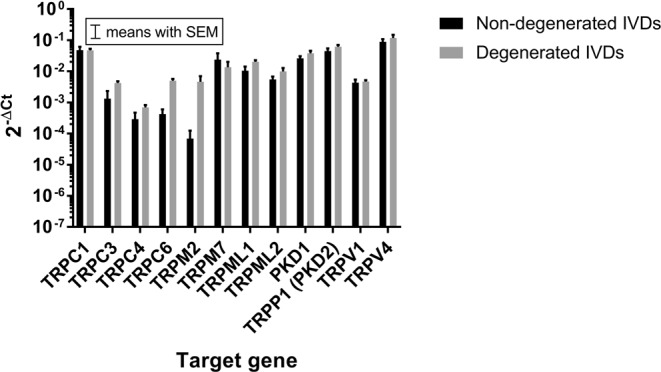
The overall gene expression of the selected TRP channels in the non-degenerated (n = 12, black bars) and degenerated (n = 22, grey bars) IVD tissue. Data presented on a logarithmic scale as 2^−ΔCt^ values (relative to YWHAZ).

Based on the gene array results, 12 targets (TRPC1, TRPC3, TRPC4, TRPC6, TRPM2, TRPM7, TRPML1, TRPML2, PKD1, TRPP1 (PKD2), TRPV1, TRPV4) were chosen for further analysis on the additional 26 IVD samples (8 non-degenerated IVDs: 4x NP and 4x AF; 18 degenerated: 8x AF, 8x NP, 2x mix tissue) using conventional qPCR (see Table 3). When combining data from all 34 samples, TRPV4 was the most highly expressed target in both non-degenerated and degenerated IVDs, followed by TRPC1, TRPP2, PKD1, and TRPM7 (Fig. 2). None of the investigated targets differed between the NP and AF zones (p > 0.05, data not shown).

**Table 3:** TaqMan primers used for the qPCR analysis.

Gene	Gene class	Primer number
YWHAZ	Internal control	Hs01122445_g1
TRPA1	Transient receptor potential channel subfamily A member 1	Hs00175798_m1
TRPC1	Transient receptor potential channel subfamily C member 1	Hs00608195_m1
TRPC2	Transient receptor potential channel subfamily C member 2	Hs03453915_g1
TRPC3	Transient receptor potential channel subfamily C member 3	Hs00162985_m1
TRPC4	Transient receptor potential channel subfamily C member 4	Hs01077392_m1
TRPC5	Transient receptor potential channel subfamily C member 5	Hs00202960_m1
TRPC6	Transient receptor potential channel subfamily C member 6	Hs00988479_m1
TRPC7	Transient receptor potential channel subfamily C member 7	Hs00220638_m1
TRPM1	Transient receptor potential channel subfamily M member 1	Hs00931865_m1
TRPM2	Transient receptor potential channel subfamily M member 2	Hs01066091_m1
TRPM3	Transient receptor potential channel subfamily M member 3	Hs00257553_m1
TRPM4	Transient receptor potential channel subfamily M member 4	Hs00214167_m1
TRPM5	Transient receptor potential channel subfamily M member 5	Hs00175822_m1
TRPM6	Transient receptor potential channel subfamily M member 6	Hs01019356_m1
TRPM7	Transient receptor potential channel subfamily M member 7	Hs00559080_m1
TRPM8	Transient receptor potential channel subfamily M member 8	Hs01066596_m1
TRPML1	Transient receptor potential channel subfamily ML member 1	Hs01100653_m1
TRPML2	Transient receptor potential channel subfamily ML member 2	Hs00401916_m1
TRPML3	Transient receptor potential channel subfamily ML member 3	Hs00539554_m1
PKD1	Polycystin 1, Transient Receptor Potential Channel Interacting	Hs00947377_m1
TRPP1 (PKD2)	Transient receptor potential channel subfamily P member 1	Hs00960946_m1
TRPP2 (PKD2L1)	Transient receptor potential channel subfamily P member 2	Hs00175850_m1
TRPP5 (PKD2L2)	Transient receptor potential channel subfamily P member 3	Hs00950467_m1
TRPV1	Transient receptor potential channel subfamily V member 1	Hs00218912_m1
TRPV2	Transient receptor potential channel subfamily V member 2	Hs00901648_m1
TRPV3	Transient receptor potential channel subfamily V member 3	Hs00376854_m1
TRPV4	Transient receptor potential channel subfamily V member 4	Hs01099348_m1
TRPV5	Transient receptor potential channel subfamily V member 5	Hs00219765_m1
TRPV6	Transient receptor potential channel subfamily V member 6	Hs00367960_m1

YWHAZ = Tyrosine 3-Monooxygenase/Tryptophan 5-Monooxygenase Activation Protein Zeta.

**Figure 3:**
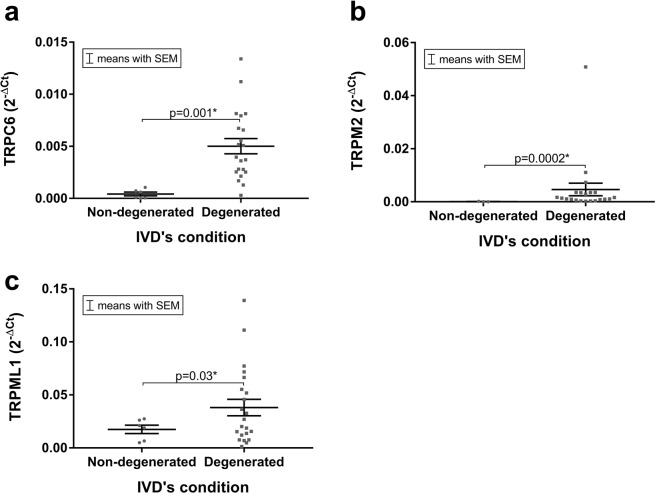
The mRNA expression of (**a)** TRPC6, (**b)** TRPM2 and (**c)** TRPML1 differed significantly between non-degenerated and degenerated human IVDs. Data presented as 2^−ΔCt^ values (relative to YWHAZ). Asterisks indicates a statistically significant difference between the groups indicated (*p < 0.05).

When comparing the expression of the TRP channels in the non-degenerated and degenerated IVDs, statistically significant differences were found in the mRNA expression levels of TRPC6 (Fig. 3a, p = 0.001), TRPM2 (Fig. 3b, p = 0.0002) and TRPML1 (Fig. 3c, p = 0.03). In all three targets, mRNA levels were higher in the degenerated IVD samples than in the non-degenerated samples.

**Figure 4:**
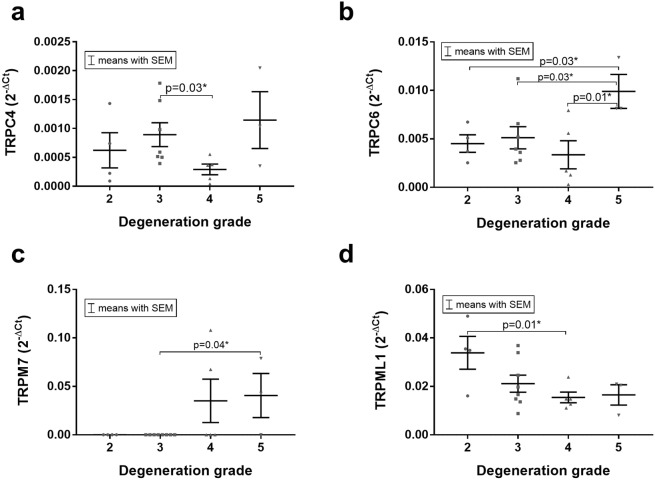
The mRNA expression of (**a)** TRPC4, (**b)** TRPC6, (**c)** TRPM7 and (**d)** TRPML1 differed significantly between different degeneration grades. Data presented as 2^−ΔCt^ values (relative to YWHAZ). Asterisks indicates a statistically significant difference between the groups indicated (*p < 0.05).

Within the degenerated IVDs (n = 22), the expression of several TRP channels was significantly affected by the degeneration grade: TRPC4 (Fig. 4a, grade 3 > 4, p = 0.03), TRPC6 (Fig. 4b, 5 > 2 p = 0.03, 5 > 3, p = 0.03, 5 > 4 p = 0.01), TRPM7 (Fig. 4c, 5 > 3, p = 0.04) and TRPML1 (Fig. 4d, 2 > 4, p = 0.01).

### TRP channel expression (mRNA): Modic changes

**Figure 5:**
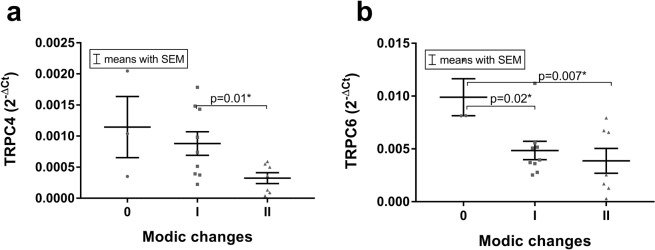
The mRNA expression levels of (**a)** TRPC4 (I > II, p = 0.01) and (**b)** TRPC6 (0 > I p = 0.02, and > II p = 0.007) were found to be affected by Modic changes (0 n = 3, I n = 10 and II n = 7). Data presented as 2^−ΔCt^ values (relative to YWHAZ). Asterisks indicates a statistically significant difference between the groups indicated (*p < 0.05).

Modic changes represent a classification of pathological changes to the endplates, where 0 indicates no pathological changes, I is characterized by a vertebral bone edema, and II by fatty replacement of the bone marrow^18^. Modic changes may be associated with the pain development^19^. We hence tested whether the presence of Modic changes affects TRP channel expression and found a significant effect for TRPC4 (Fig. 5a, I > II, p = 0.01) and TRPC6 (Fig. 5b, 0 > I p = 0.02, 0 > II p = 0.007).

### TRP channel expression (mRNA): Pain intensity, pain duration and steroid use

**Figure 6:**
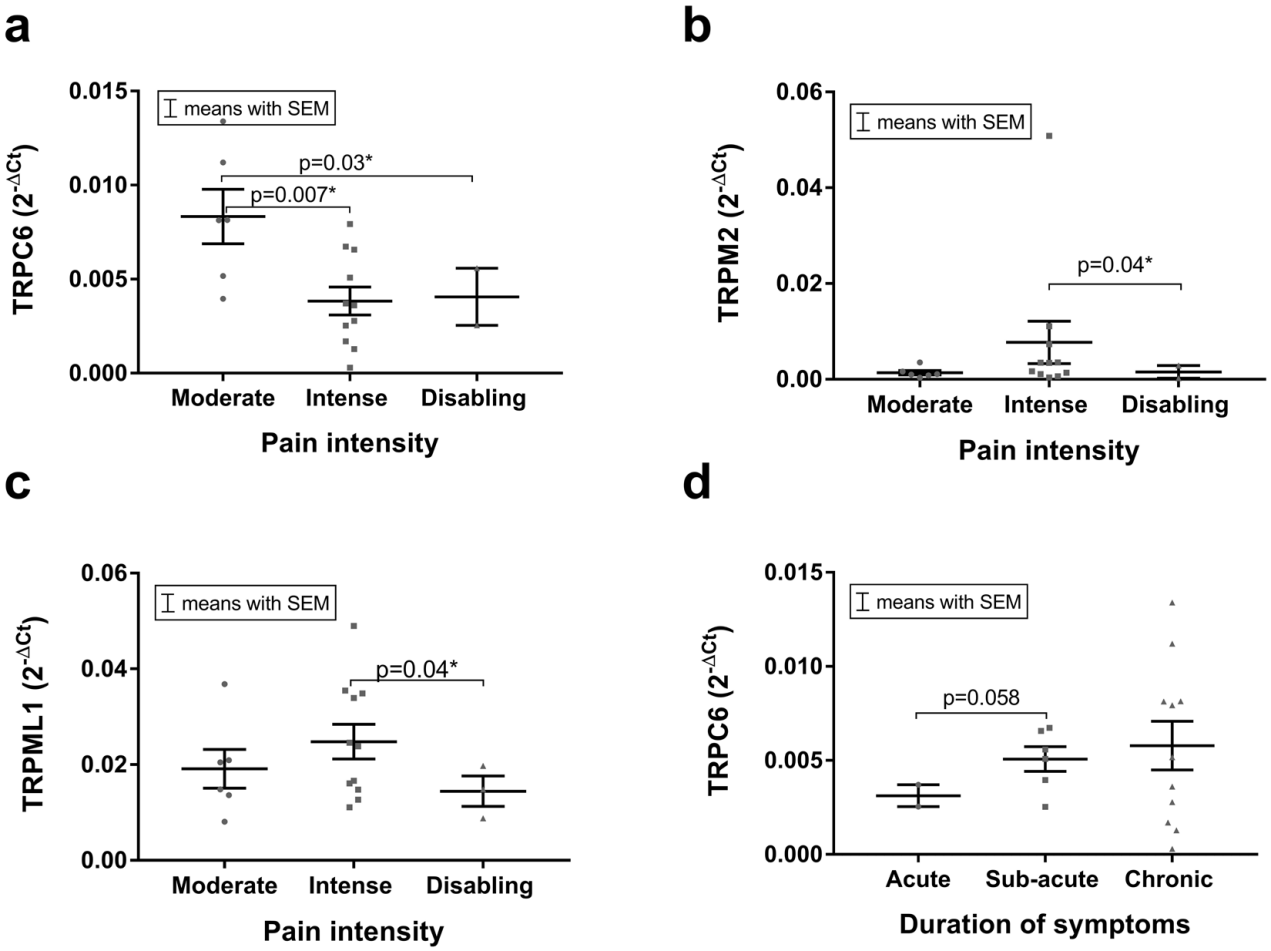
The mRNA expression of (**a)** TRPC6 was significantly higher in the moderate pain group compared to the intense (p = 0.007) and disabling (p = 0.03) pain groups, whereas the mRNA expression of (**b)** TRPM2 and (**c)** TRPML1 was statistically different between the intense and disabling pain groups (p = 0.04). Although no statistical differences were found between the duration of symptoms (acute: >2 months, sub-acute: 2–12 months, and chronic: >1 year), (**d)** the TRPC6 mRNA expression tended to increase with the duration of pain symptoms. Data presented as 2^−ΔCt^ values (relative to YWHAZ). Asterisks indicates a statistically significant difference between the groups indicated (*p < 0.05).

Degenerated IVD samples obtained from patients who assessed their pain as moderate were characterized by significantly higher TRPC6 expression levels compared to those obtained from patients with intense pain (p = 0.007, Fig. 6a) or disabling pain (p = 0.03, Fig. 6a). Interestingly, TRPC6 expression tended to increase with the duration of pain symptoms, but no statistical significance was found between the groups (p = 0.058, Fig. 6d). Expression of TRPM2 and TRPML1 was found to be the highest in the IVDs obtained from patients who defined their pain as intense and was found to be statistically different from the IVDs obtained from patients with disabling (TRPM2 p = 0.04 Fig. 6b, TRPML1 p = 0.04 Fig. 6c).

**Figure 7:**
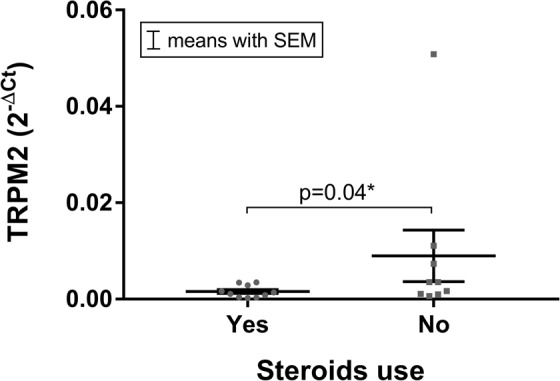
The mRNA expression of TRPM2 was significantly lower (p = 0.04) in patients who received a steroid treatment. Data presented as 2^−ΔCt^ values (relative to YWHAZ). Asterisks indicates a statistically significant difference between the groups indicated (*p < 0.05)

In addition, TRPM2 mRNA expression was significantly decreased in IVD samples obtained from patients who received steroid treatment (p = 0.04, Fig. 7).

### TRP channels protein expression: Immunohistochemistry

In the next step, TRPC6, TRPM2 and TRPML1 expression changes observed during degeneration progression on the mRNA level (Figs. 2, 3a–c, 4b,d, 5b, 6a–d and 7) were investigated on the protein level by immunohistochemistry. A total of seven surgical symptomatic degenerated IVD tissue samples, two non-degenerated young IVD samples and one aged asymptomatic (non-painful) IVD sample with mild multifocal degenerative changes were examined per target. Details regarding patient information and a complete evaluation, as well as the applied scoring system can be found in Tables 4–7, respectively.

**Table 4:** Immunohistochemistry (IHC): patient information in relation to clinical findings.

Sample No.	Condition	Region	Age	Sex	Pathology	Degeneration grade*	Modic type	TRPC6	TRPM2	TRPML1
35	1	AF	76	m	DDD	3	2		x	x
36	1	NP	47	m	DH	3	1		x	x
37	1	mix	59	f	DH	3	1		x	x
38	1	AF	56	f	DDD	3	n/a		x	x
39	1	AF	43	f	DDD	3	2		x	x
40	1	mix	32	f	DDD	3	1	x		
41	1	mix	50	f	DDD	3	2	x		
42	1	mix	47	m	DDD	3	1	x		
43	1	mix	74	m	DDD	3	2	x		
44	1	NP	66	m	DH	3	1	x		
45	1	mix	67	m	DDD	4	2	x	x	x
46	1	mix	71	f	DDD	4	2	x	x	x
47	0	entire IVD	16	f	#	2	#	x	x	x
48	1	entire IVD	77	f	n/a	4	n/a	x	x	x
49	0	entire IVD	3 days	m	#	1	#	x	x	x

1 = degenerated; 0 = non-degenerated; NP = nucleus pulposus; AF = annulus fibrosus; m = male; f = female; DH = disc herniation; DDD = degenerative disc disease; n/a = data not available; # = not applicable; * cadaveric samples were assessed with Thompson classification and degenerated biopsies with Pfirrmann classification; x = sample on which IHC was performed with a listed antibody.

**Table 5:** Immunohistochemistry (IHC): patient information in relation to clinical findings.

Sample No.	BMI^#^	Steroids^#^	Smoking^#^	Pain score^#^	Duration of symptoms (grouped) ^#^	Cause of death*	TRPC6	TRPM2	TRPML1
35	27.8	0	0	2	2			x	x
36	26.9	0	0	3	2			x	x
37	33	1	1	2	2			x	x
38	28	1	0	3	2			x	x
39	26	1	1	2	2			x	x
40	23.8	0	1	1	3		x		
41	24.4	1	1	3	3		x		
42	33	0	1	1	3		x		
43	28	1	0	2	3		x		
44	26	1	1	2	3		x		
45	25	0	0	2	2		x	x	x
46	20	0	1	2	2		x	x	x
47						craniocerebral trauma	x	x	x
48						myocardial infarction	x	x	x
49						endocarditis	x	x	x

Steroids or smoking: 0 = no, 1 = yes; Pain score: 1 = moderate pain, 2 = intense pain, 3 = disabling pain; Pain duration: 1: 1–2 months, 2: 2–12 months, 3: > 1 year; * = applicable only to the entire IVD samples, donors 47 to 49; # = not applicable to the entire IVD samples, donors 47 to 49; x = sample on which IHC was performed with a listed antibody.

**Table 6:** Immunohistochemistry (IHC): Evaluation.

Sample No.	Condition^*^	Region	Expression grade (IHC signal intensity)			Distribution (% positive cells)			Total score (A + B)		
			TRPC6	TRPM2	TRPML1	TRPC6	TRPM2	TRPML1	TRPC6	TRPM2	TRPML1
35	1	AF		1	1		5	1		6	2
36	1	NP		2	1		4	3		6	4
37	1	mix		1	3		4	3		5	6
38	1	AF		2	1		5	2		7	3
39	1	AF		2	2		5	3		7	5
40	1	mix	3			5			8		
41	1	mix	2			5			7		
42	1	mix	1			1			2		
43	1	mix	2			3			5		
44	1	NP	2			2			4		
45	1	mix	3	3	1	5	5	4	8	8	5
46	1	mix	3	3	3	5	5	4	8	8	7
47	0	entire IVD	0	0	0	0	0	0	0	0	0
48	1	entire IVD	2	2	1	3	3	1	5	5	2
49	0	entire IVD	0	1	1	0	3	1	0	4	2

1 = degenerated; 0 = non-degenerated; x = sample on which IHC was performed with a listed antibody; scoring system is described in Table 5.

**Table 7:** Combinative semi-quantitative scoring system by Fedchenko and Reifenrath^48^.

Proportion score A	Positive cells, %	Signal intensity	Intensity score B
0	0	None (−)	0
1	<1	Weak (+)	1
2	1 to 10	Intermediate (++)	2
3	11 to 33	Strong (+++)	3
4	34 to 66	**Final score (A + B): 0–8**	
5	≥67		

**Figure 8:**
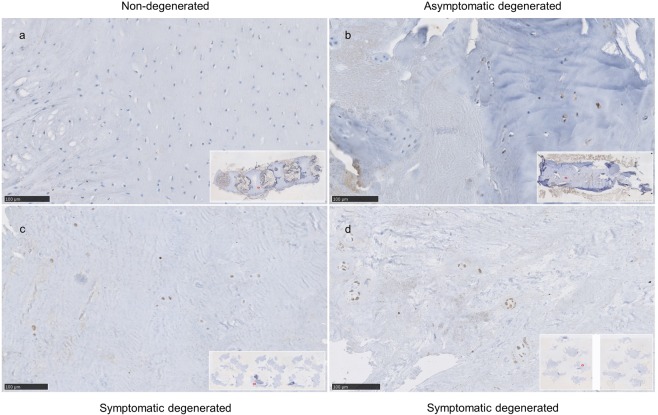
Immunohistochemical staining against TRPM2 in (**a)** non-degenerated IVD section obtained from a young donor (n.49) and (**b)** asymptomatic (non-painful) IVD section with mild multifocal degenerative changes (n.48) showing weak staining positivity as compared to (**c,d**) symptomatic degenerated IVD sections obtained from patients undergoing a low back surgery (**c**, n.37, Pfirrmann degeneration grade = 3) and **(d**, n.46, degeneration grade = 4). Scale bar is 100 µm.

TRPM2 protein signal intensity was weak or absent in young non-degenerated IVD samples (Fig. 8a) and it was increased in asymptomatic (Fig. 8b) and symptomatic (Fig. 8c,d) degenerated IVD samples, where it reached the highest average combined score (6.71 ± 1.11) out of the three investigated targets. Overall, in the symptomatic degenerated IVD samples, more positively stained cells were detected as compared to the asymptomatic aged and young IVD samples (4.71 ± 0.49 vs. 2.0 ± 1.73). Taken together, these findings reflect degeneration-driven expression pattern observed earlier on the gene level (Figs. 2 and 3b).

**Figure 9:**
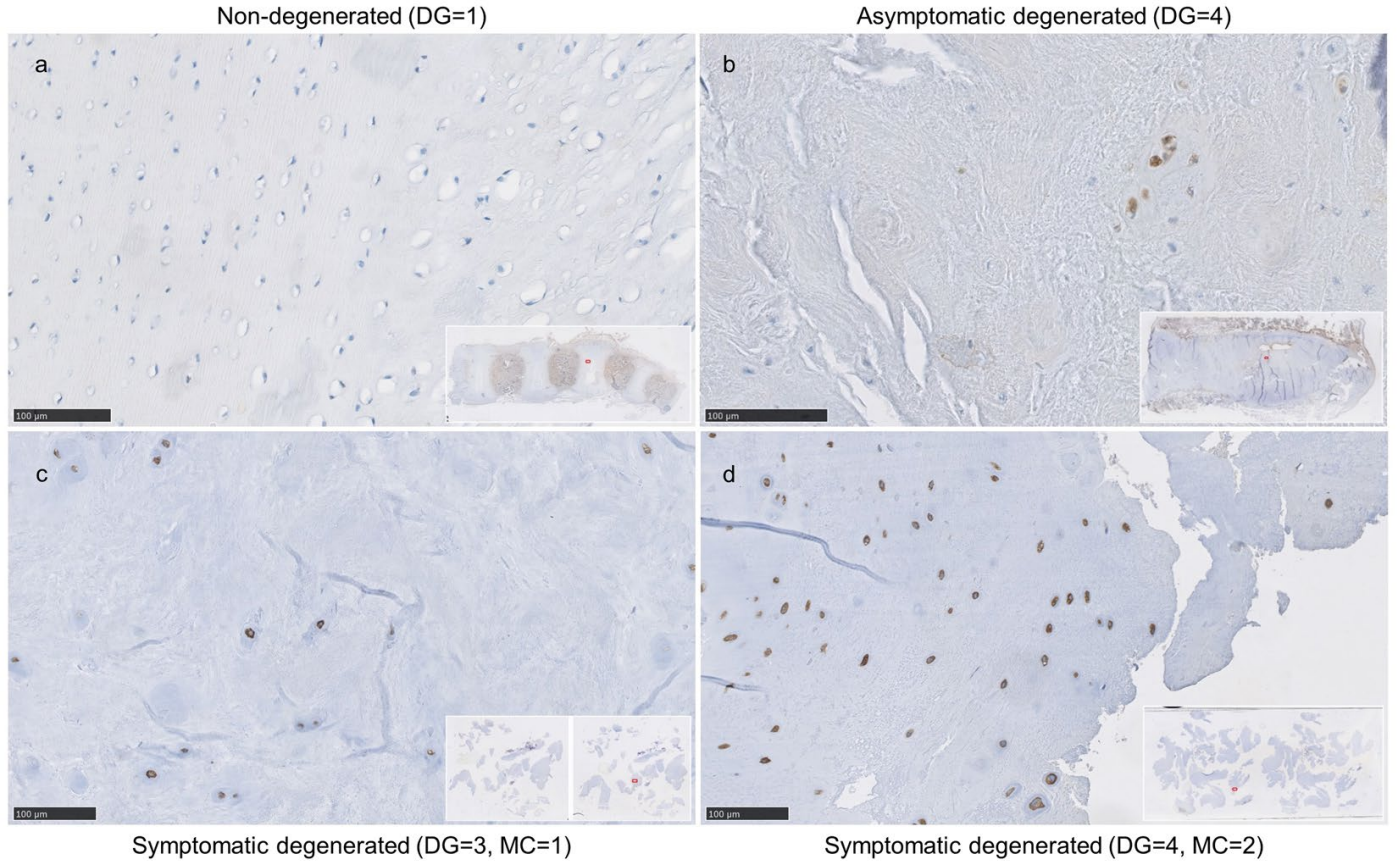
Immunohistochemical staining against TRPC6 in (**a)** non-degenerated IVD section obtained from a young donor (n.49) presenting a TRPC6 protein expression. IVD sections illustrating increased expression with age and degeneration: (**b)** asymptomatic (non-painful) IVD section with mild multifocal degenerative changes (n.48), (**c,d)** symptomatic degenerated IVD sections obtained from patients undergoing low back surgery (**c**, n.40, Pfirrmann degeneration grade (DG) = 3, Modic changes (MC) = 1) and (**d**, n.45, Pfirrmann degeneration grade (DG) = 4, Modic changes (MC) = 2). Cadaveric samples were assessed with Thompson classification and degenerated biopsies with Pfirrmann classification. Scale bar is 100 µm.

TRPC6 protein expression levels were overall comparable to TRPM2, thus reflecting our gene expression data (Fig. 2). While no expression was observed in non-degenerated young samples (Fig. 9a), the observed staining intensity was similarly high in symptomatic degenerated IVD samples, showing a moderate signal positivity in up to 20% of the cells, and the asymptomatic aged sample (Fig. 9b–d), presenting moderate to strong positivity in most of the cells. In the examined symptomatic degenerated IVD tissue samples, TRPC6 was highly expressed in 6 out of 7 samples (6.00 ± 2.38), with an expression pattern comparable to the TRPC6 gene expression results on degeneration grade and Modic score (Figs. 3a and 5b). One sample was considered to be negative for the TRCP6 protein expression with the overall score value 2, which was a result of lower samples quality with insufficient amount of preserved tissue structure.

**Figure 10:**
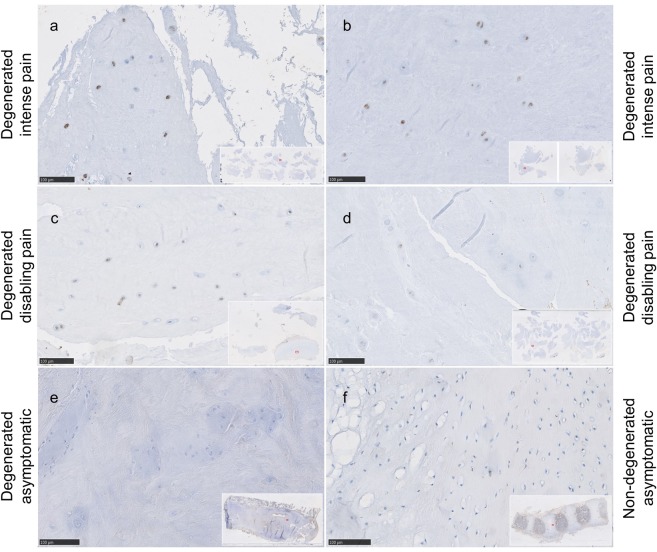
Immunohistochemical staining against TRPML1 in symptomatic degenerated IVD sections with high TRPML1 staining intensity in (**a,b)** obtained from patients suffering from intense low back pain (n.37 and n.39 respectively) as compared to sections with moderate staining intensity (**c,d)** obtained from patients suffering from disabling low back pain (n.36 and n.38 respectively); and sections negative for TRPML1: (**e**) asymptomatic (non-painful) IVD section with mild multifocal degenerative changes (n.48) and **f)** non-degenerated IVD (n.49). Scale bar is 100 µm.

TRPML1 protein expression was weak to absent in asymptomatic and non-degenerated IVD samples (Fig. 10e,f). In symptomatic degenerated IVD samples, TRPML1 protein expression was observed in 6 out of 7 cases (Fig. 10a–d), with an average final score of 4.57 ± 1.72, and was characterized by weak to moderate signal positivity and mostly in areas, where clusters of cells were present. One sample was negative for TRPML1 presenting only single cells with a faint signal (score 2). In general, the TRPML1 signal intensity was weaker compared to TRPC6 and TRPM2, and the positivity was limited to a lower number of cells. In contrast, on the gene level TRPML1 expression was slightly higher than TRPC6 and TRPM2 expression (Fig. 2). Within the symptomatic degenerated IVD samples, TRPML1 tended to be expressed overall higher in samples collected from patients experiencing intense pain (Fig. 10a,b) in comparison to disabling pain (Fig. 10c,d), and asymptomatic and non-degenerated samples (Fig. 10e,f), hence illustrating a similar pattern as observed on the gene level (Fig. 6c).

## Discussion

Degeneration of the intervertebral disc is one of the leading causes of low back pain, which not only lessens the patients’ quality of life, but also constitutes a high financial burden on the society. With the aging population, the occurrence as well as costs of IVD degeneration and LBP are predicted to grow. Hence, it is crucial to gain a better insight into the molecular processes of IVD degeneration along with a potential to develop new and targeted treatment strategies. Recently, TRP channels, which constitute a family of ion channels that were shown to play an important role in various diseases^20^, have emerged as potential targets^10^, but their exact function in the IVD remains unknown. This study is the first one to report a complete screening of TRP channels in non-degenerated and degenerated IVDs. Our results confirm the important role of the TRPC subfamily in the IVD, but also highlight previously unexplored targets stemming from e.g. the TRPM or TRPML subfamily.

According to our results and in line with previously reported findings^13^, the highest observed expressed TRP channel overall in the IVD is TRPV4. However, no significant differences were found for any of the analyzed patient/tissue factors for TRPV4. Similarly, TRPV1 was well expressed in both non-degenerated and degenerated IVD tissue, but no significant differences were found when evaluated against patient- or tissue-related factors. TRPV1 is responsive to pro-inflammatory agents (e.g. TNF-α), which are known to be higher expressed in painful IVD tissue. Therefore, the inflammatory environment associated with DDD likely increases the probability for channel opening^21^. Other members of the TRPV channel family, but TRPV1 and TRPV4, where not detected or were expressed at the detection limit during the initial screening and were hence excluded from the further analysis in this study. The TRP vanilloid (V) subfamily constitutes of six members (V1–6) in humans, with a main role in thermo-sensation. Additionally, TRPV4 was shown to also play a role in mechano- and osmo-sensation in the IVD and cartilage^22^ and TRPV1 may be gated by low pH (≤5.9)^20^. Upon channel activation (via e.g. mechanical or osmotic stress), TRPV4 causes transient increases of intracellular calcium, which acts as a secondary messenger that activates NFAT5/TonEBP, MAP kinases and nuclear factor kappa-light-chain-enhancer of activated B cells (NF-κB) pathways^23^, which are involved in cell hemostasis as well as inflammation^24^. It was demonstrated that TRPV4 expression increases with decreasing IVD osmolarity as observed during IVD degeneration^9^ and may correlate with pro-inflammatory molecules such as TNF-α^9^ and interferons (IFN) A1 and B1^13^ in the degenerated IVD. Interestingly, TRPV1 was shown to function as an osmosensor in the brain^25,26^, although no supporting data exists on such function in the IVD. Taken together, since TRPV1 and TRPV4 are expressed in both non-degenerated and degenerated IVD tissue, it may indicate that both channels are important for the IVD cells fundamental functions. However, future studies should confirm their exact function and further analyze the previously reported involvement of TRPV4 in IVD degeneration^9^.

In contrast to TRPV4 and TRPV1, the expression of TRPC6 was broadly affected by patient-related characteristics. TRPC6 belongs to the TRP canonical (C) subfamily, together with five other channels (C1–6). The TRPC subfamily may be involved in mechanosensing^14,22^, as well as in pain and inflammation in joint tissues^10^. We were able to show that the mRNA expression of TRPC6 was significantly higher in degenerated IVDs than in non-degenerated IVDs and that its expression tended to increase with the IVD degeneration grade as previously reported^13^. In line with it, on the protein level, TRPC6 expression was consistent in the asymptomatic and symptomatic degenerated IVD samples and it was absent in non-degenerated IVD samples. Moreover, we showed that higher TRPC6 gene expression was associated with specific Modic types, moderate pain and tended to increase with the duration of disease symptoms. Due to limited sample size, we were not able to confirm these findings on the protein level. Interestingly, no zonal (AF, NP) differences were found (data not shown) in contrary to a previous publication^13^. However, this may be due to the fact that a clear separation of the zones within IVD tissue (especially in higher stages of degeneration) is difficult, as well as due to a smaller sample size. TRPC6 was previously shown to correlate with inflammatory markers such as TNF-α, IL-6, IL-8 and IL-15 in the IVD^13^ and potentially plays a significant role in modulating immune response, as well as mediate inflammatory processes in other tissue types such as lung, kidney and neurons^27^. These data indicate that TRPC6 could be an interesting pharmaceutical target for pain treatment of patients with high degree of disc degeneration.

Besides TRPC6, three other members of the canonical subfamily, TRPC1, TRPC3 and TRPC4, were screened in this study. TRPC1 and TRPC3 were detected at similar levels in non-degenerated and degenerated IVDs and they showed no statistically significant relation to any of investigated factors. Both channels were previously detected on the mRNA, but not on the protein level in human chondrocytes, where elevated TRPC3 mRNA expression levels, but not TRPC1 were observed with higher passaging^25^. TRPC1 and TRPC3 are also hypothesized to have mechanosensitive function, but their exact role in the IVD remains thus far unknown^10^. For the last investigated canonical member, TRPC4, we could show differences in its expression with the degree of IVD degeneration and the type of Modic change. Although previously unexplored in the IVD, TRPC4 is known to be expressed in human chondrocytes^25^ as well as in the central nervous system (CNS), smooth muscle cells, kidney and endothelium^22^. In the CNS, it contributes to the axonal regeneration after the dorsal root ganglion injury^28^ and hence may be involved in the regulation of IVD innervation.

The TRPM2 and TRPM7 channels belong to the TRP melastatin (M) subfamily that consists of eight members (M1–8) in humans and is most known for its function in thermo-sensation^22^. Importantly, TRPM7 was also detected in human chondrocytes, where it showed the overall strongest mRNA expression out of all tested target^25^. Similarly as in the human chondrocytes^25^, TRPM7 was overall consistently expressed in non-degenerated and degenerated human IVDs, albeit with higher expression in IVDs with higher degeneration grades. Although no other data on TRPM7 in the IVD exist, it was shown that TRPM7 may act as a mechano-sensor and mediate osmolarity-induced cell volume changes in the kidney and salivary glands^29,30^. A non-degenerated IVD tissue is characterized by diurnal osmolarity changes (~400–500 mOsm) while reduced tissue osmolarity (~300 mOsm) is a hallmark of IVD degeneration^31^. Since an osmotic balance is necessary for an IVD to maintain its function, it could be hypothesized that TRPM7 may be involved in osmo-sensing and osmo-adaptation in the IVD.

TRPM2 is hypothesized to play an important role in pain development by acting as an oxidative stress and reactive oxygen species (ROS) sensor in immune cells^32^ and regulator of pro-inflammatory cytokine release in the CNS^33^. In this study, TRPM2 was expressed at higher mRNA levels in degenerated IVDs compared to non-degenerated IVDs. Additionally, in the non-degenerated IVD samples, TRPM2 was detected only in the AF tissue, whereas in the degenerated IVDs, TRPM2 was detected both in the AF and NP tissue. These data imply that TRPM2 exists in a native human AF tissue, however its elevated levels, as well as its occurrence in both AF and NP tissue may be due to the nerve and blood vessels ingrowth, which are commonly observed during IVD degeneration^34^, as well as to a possible contamination during tissue separation. On the protein level, TRPM2 was consistently well expressed in symptomatic and asymptomatic degenerated IVD samples and to the lesser extend or not at all in the non-degenerated samples, hence pointing towards degeneration-driven TRPM2 expression. Interestingly, we could show that TRPM2 mRNA expression was strongly linked to pain. We could also demonstrate that the TRPM2 mRNA expression was significantly decreased in patients, who underwent steroid treatment and was higher in patients who described their pain as intense. However, it should be noted that the majority of patients who described their pain as disabling were also the ones who received steroid treatment. These data indicate a pivotal role of TRPM2 in pain mediation in the IVD and suggest that its modulation may create new opportunities for therapeutic strategies of the low back pain treatment.

The TRP mucolipin (ML) subfamily consists of three, thus far poorly characterized, members (ML1–3) in humans. Recent evidence suggest that TRPML channels may be involved in the lysosomal function and regulation of autophagy^22,35^, which can be also present in the IVD. Increased TRPML1 activity was observed during stress conditions and can be triggered by catabolic signaling^36^. Shen *et al*.^37^ showed serum deprivation and inflammation induced autophagy of rat AF cells. In contrast, compression and ROS induced generation of autophagy in rat NP cells^38^ and reduced the catabolic effects of IL-1B and TNF-α^39^. However, whether and how TRPML channels are involved in autophagy in the IVD is unknown. Here, we could show for the first time that the TRPML subfamily is present in the IVD, with TRPML1 being significantly higher expressed on the gene level in the degenerated IVDs, in an inverse, grade-depended manner. On the protein level, TRPML1 was detected in symptomatic degenerated IVDs, but not in non-degenerated and asymptomatic (non-painful) IVD samples. Additionally, we could show that the expression of TRPML1 on gene and protein level was higher in patients who described their pain as intense.

The TRP polycystin (P) subfamily compromises three channel members (TRPP1 known as PKD2 (previously TRPP2), TRPP2 known as PKD2L1 (previously TRPP3) and TRPP3 known as PKD2L2 (previously TRPP5)^40^. Additional information on the TRPP channel subfamily nomenclature is provided in the Supplementary Material. Importantly, TRPP1 (PKD2) does not produce cation currents until assembled with PKD1^41^. Although we were able to detect PKD1 and TRPP1 (PKD2) in non-degenerated and degenerated specimens at relatively high levels (yet unaffected by patient- or tissue-related characteristics), the lack of general information on their function hinders interpretation of their relevance in the IVD. It was proposed that TRPP1 (PKD2) might either be a mechanosensitive channel or regulate mechanosensitive channels by interacting with the TRPV4 in the cilium^40,42^ and rat endothelial cells^43^.

Although TRP channels constitute potential pharmaceutical targets for the treatment of disc-related back pain and IVD degeneration, their modulation has to be approached with caution as several TRP channels are also present in non-degenerated IVDs and therefore are likely to be of relevance not only in tissue pathophysiology, but also homeostasis. Therefore, the channel activity in non-degenerated and degenerated IVDs should also be considered and determined in order to design adequate treatment strategies such as pharmacological inhibition of TRP channels. In recent years, several potent and selective TRP channels agonists and antagonists have been identified (e.g. GSK1016790A activates and GSK2193874 blocks TRPV4, Waixenicin A inhibits TRPM7 etc.), however many compounds commonly used in TRP channel research also activate/inhibit a broad range of other channels (e.g. SKF96365 can inhibit TRPC subfamily, as well as TRPV4)^44–46^. Future developments of specific modulators of those TRP channels potentially involved in back pain may constitute a viable alternative to intradiscal steroid injections (with high side effects) or spinal surgery (with high complication rates). Furthermore, future studies should determine whether enhanced mRNA and protein expression in degenerated samples (e.g. for TRPC6, TRPM2 and TRPML1) is a protective mechanism or de facto contributes to the progression of IVD degeneration. Clear technical limitations of this study include a small sample size, especially of non-degenerated samples that are rare to obtain. Likewise, a bigger sample size could allow for a more comprehensive screening of selected targets on the protein level.

This study is the first to present a complete screening of all currently known TRP channels in non-degenerated and degenerated IVD tissue. In summary, our results indicate that TRP channels may play an important role in IVD degeneration, as well as in low back pain. We were able to confirm the presence and relevance of previously described TRP channels (e.g. TPC6) in degenerated IVDs, but also reveal novel TRP candidates originating e.g. from the TRPM, TRPML and TRPP subfamilies. These findings may be of importance in designing future anti-pain treatment strategies, as well as for the better understanding of the molecular mechanisms underlying disc degeneration and degenerative disc disease.

## Materials and Methods

### Sample collection

A total of 34 IVD samples were obtained from 27 individuals (mean age = 49.7 [age range 17–80 years]):

- Twenty individuals underwent elective spinal surgery due to IVD degeneration/herniation (=22 degenerated IVD samples). Informed consent for sample collection was obtained from each patient and the study was approved through the local ethics committee (Ethics Committee of the Canton Lucerne/Switzerland, #1007).
- Seven individuals were organ donors without any signs of IVD degeneration and no history of back pain (=12 non-degenerated IVD samples). Informed consent for sample collection was obtained from family members and the study was approved through the local ethics committee (A04-M53-08B). Spines were harvested within 3 h of the aortic clamping.

Following macroscopic tissue evaluation, each sample was characterized as NP (degenerated n = 10, non-degeneratedd n = 7) and/or AF (degenerated n = 10, non-degeneratedd n = 5) whenever possible. Samples in which no distinction was possible were termed mix (degenerated n = 2). Assessment of the disease state was performed using Pfirrmann grading (IVD degeneration) and Modic grading (endplate changes) for the degenerated samples, as well as Thompson grading for the organ donor samples. Detailed patient information is given in Tables 1 and 2. All methods were performed in accordance with institutional guidelines and regulations.

### RNA extraction from tissue

IVD tissue samples were saved in RNAlater (Thermo Fisher, Switzerland) and transported to the laboratory. Upon processing, RNAlater was aspirated and tissue samples were shock frozen in liquid nitrogen, and pulverized using custom-made grinders. The obtained tissue powder was transferred into TRIzol (1 ml per 200 mg tissue, Thermo Scientific, Switzerland) and the sample was further homogenized with a polytron three times for 30 seconds (POLYTRON® PT 10/35 GT), with cooling on ice in between. Next, samples were incubated for 5 min at RT, vortexed and centrifuged (4 °C, 12000 g, 15 min) to remove tissue debris. Supernatants were supplemented with chloroform (1 part chloroform to 5 parts sample), vortexed and incubated for 5 min at RT. Next, samples were centrifuged (4 °C, 12000 g, 10 min), the aqueous phase was mixed with 70% ethanol (1:1 ratio) and RNA was purified by the RNeasy Mini Kit (Qiagen, Switzerland), according to the manufacturer’s recommendation. The quality and quantity of RNA was quantified using a Nanodrop (Thermo Fisher, Switzerland), specifically controlling the 260/280 and 260/230 ratio.

### RNA extraction from isolated IVD cells

Five out of 22 degenerated IVD samples with sufficient size were not only used for direct RNA isolation as described above, but also for IVD cell isolation in order to test whether enzymatic digestion affects the TRP channel expression profile (see Supplementary Material).

### cDNA synthesis and pre-amplification

Extracted RNA was reverse transcribed into a cDNA in a total volume of 60 µL, using the reverse transcription kit (Thermo Fisher, Switzerland). Subsequently, a preamplification step with the TaqMan PreAmp Master Mix (2×) (Thermo Fisher, Switzerland) and Custom TaqMan PreAmp Pools (Thermo Fisher, Switzerland) was conducted according to the manufacturer’s protocol. Briefly, 100 ng of cDNA in 5 µL total volume was mixed with 5 µL of Custom TaqMan PreAmp Pool and 10 µL of TaqMan PreAmp Master Mix (2×), followed by a pre-amplification through 14 cycles at 60 °C/90 °C.

### Gene expression analysis: Array

In order to identify the most prominent TRP channel candidates in the IVD, an initial set of eight IVD samples was used for the gene expression screening with TaqMan Array Fast Plates (Thermo Fisher, Switzerland). The screening sample set included four degenerated IVD samples (1x AF Pfirrmann Grade 4, 1x NP Pfirrmann Grade 4, 1x AF Pfirrmann Grade 5, 1x NP Pfirrmann Grade 5) and four non-degenerated IVD samples (2x AF, 2x NP).

The array was conducted according to the protocol provided by the manufacturer. Briefly, 180 µL of amplified cDNA (mixed with RNAse-free water) was combined with 180 µL of the TaqMan Fast Universal PCR Master Mix (2×) (Thermo Fisher, Switzerland) and added to a 96-well plate (10 µL per well), pre-coated with selected TaqMan primers, and gene expression was measured by the real-time qPCR (CFX96 Touch™ Detection System, Biorad). Each array constituted of 32 targets out of which 28 included human TRP channel targets: TRPA1, TRPC1–7, TRPM1–8, TRPML1–3, TRPP1–3, TRPV1–6, and PKD1, as well as three internal controls: 18S, YWHAZ and GUSB. Additionally, to identify the most stable reference gene, six additional internal controls (ACTB, GAPDH, RPL4, RPL13A, SDHA and TBP, see Supplementary Material) were tested on the IVD screening sample set and analyzed with the geNorm algorithm^47^.

### Gene expression analysis: standard qPCR

The remaining samples underwent standard qPCR (CFX96 Touch™ Detection System, Biorad, Hercules, CA, USA), measuring all selected TRP channels as well as YWHAZ in duplicates. To ensure comparability with the array data, identical TaqMan primers (Table 3) were used.

The qPCR and the array data were combined and the obtained Ct values were analyzed by comparative method (gene of interest relative to YWHAZ) and displayed as 2^−ΔCt^ values. To determine whether TRP channels gene expression was affected by enzymatic cell isolation, expression in cells was compared to its respective tissue sample and results are displayed as 2^-ΔΔCt^ (see Supplementary Material).

### Immunohistochemical (IHC) analysis of TRPC6, TRPM2 and TRPML1 expression in human IVD tissue

Immunohistochemistry specific for TRPC6, TRPM2 and TRPML1 was performed on 3 μm histologic sections cut from paraffin embedded IVD tissue samples that were excised from patients undergoing spinal surgery due to back pain (n = 7 for each marker). Additional asymptomatic IVD sections (n = 3) were added for each marker (detailed patient information given in Tables 4 and 5). All sections were deparaffinized. For TRPM2, heat-mediated antigen retrieval (0.1 M citrate puffer, ph 6.0) was used for 20 min (97 °C, Agilent Dako PT-Link Pre-Treatment Module), while all other antibodies were “ready-to-use”. The endogenous peroxidase was blocked with 0.2% sodium azide and 3% hydrogen peroxide; the non-specific protein was bound by “Protein Block Serum-Free” (Agilent Dako X0909 Lot#1014188). Thereafter, sections were incubated with rabbit polyclonal anti-TRPC6 (1:50, Alomone, #ACC-120, lot: ACC120AN0350), rabbit polyclonal anti-TRPM2 (1:50, Abcam, ab11168, lot: GR30307-24) or rabbit polyclonal anti- Mucolipin 1 (TRPML1, 1:300, Osenses, OSM00039W, lot: Rb0064-180807-WS) overnight at room temperature. Secondary detection was performed with goat anti-mouse and anti-rabbit immunoglobulins IgG (Dako, EnVisionTM + Dual Link System-HRP) for 30 min at room temperature. The amino-9-ethyl-carbazole substrate kit (Dako) was employed as a chromogen. Finally, the sections were counter-stained with Gill’s hematoxylin for 3 min, and cover-slipped with an aqueous mounting media (Glicerine, Sigma-Aldrich). The evaluation was performed on the Leica DMR System with Leica DFC320 Camera (Leica Microsystems) and compared with image scans. The images were scanned using NanoZoomer S360 Digital slide scanner: C13220-01 and extracted with NDP.view2 Viewing software. All slides were examined and semi-quantitatively evaluated by two investigators for percentage of positively stained cells and the intensity of the reaction product (visible staining). The two scores (A,B) were added together for a final score with eight possible values. Scores of 0 to 2 were considered negative and scores of 3 to 8 were considered positive (Table 7). The complete IHC evaluation is presented in Table 6.

### Statistical analysis

Data consistency was checked and data were screened for outliers. Continuous variables were also tested for normality by using skewness, kurtosis,and omnibus test. Variance homogeneity between both groups were tested by using variance ratio test and modified Levene test. In case of deviation of normality, randomization tests were computed based on Monte Carlo simulations (due to the small sample sizes). In case of normality, Student t-tests for equal or Aspin-Welch unequal variance tests were applied. All reported tests were two-sided, and p-values < 0.05 were considered as statistically significant. All statistical analyses in this report were performed by use of NCSS (NCSS 10, NCSS, LLC. Kaysville, UT) and STATISTICA 13 (Hill, T. & Lewicki, P. Statistics: Methods and Applications. StatSoft, Tulsa, OK). All figures were created using GraphPad Prism version 7.03 for Windows, GraphPad Software, La Jolla California USA.

### Ethical approval

All procedures performed in studies involving human participants were in accordance with the ethical standards of the institutional and/or national research committee and with the 1964 Helsinki Declaration and its later amendments or comparable ethical standards.

## Supplementary information

Supplementary Material

## Data Availability

The datasets generated and analyzed during this study are available from the corresponding author on a reasonable request.
