# Supplementary material for: Differential regulation of TRP channel gene and protein expression by intervertebral disc degeneration and back pain
Source: Sci Rep. 2019 Dec 11;9:18889. doi: 10.1038/s41598-019-55212-9 (PMC6906425; doi:10.1038/s41598-019-55212-9)
Supplement: Supplementary file 1 — Supplementary Material [file 41598_2019_55212_MOESM1_ESM.pdf]

Supplementary Material

Differential regulation of TRP channel gene and protein expression by intervertebral disc degeneration and back pain

A. Sadowska[1], W. Hitzl[2,3,4], A.Karol[5], P. Jaszczuk[6], H. Cherif[7,8,9], L. Haglund[7,8,9], O. N. Hausmann[6#], K. Wuertz-Kozak*[#1,10,11,12]

1. Institute for Biomechanics, D-HEST, ETH Zurich, Zurich, CH
2. Research Office - Biostatistics, Paracelsus Medical University, Salzburg, AT
3. Department of Ophthalmology and Optometry, Paracelsus Medical University, Salzburg, AT
4. Research Program Experimental Ophthalmology and Glaucoma Research, Paracelsus Medical University Salzburg, AT
5. Musculoskeletal Research Unit (MSRU), Department of Molecular Mechanisms of Disease (DMMD), Vetsuisse Faculty, University of Zurich, Zurich, CH
6. Klinik St. Anna-Hirslanden, Neuro- and Spine Center, Lucerne, CH
7. Orthopaedic Research Laboratory, Montreal, QC, CA
8. McGill Scoliosis and Spine Research Group, Montreal, QC, CA
9. The Shriners Hospital for Children, Montreal, QC, CA
10. Academic Teaching Hospital and Spine Research Institute, Paracelsus Medical University, Salzburg, AT
11. Spine Center, Schön Clinic Munich Harlaching, Munich, DE
12. Department of Health Sciences, University of Potsdam, Potsdam, DE

* Corresponding author: Karin Wuertz-Kozak, ETH Zurich, Hoenggerbergring 64, HPP-O12, Zurich, 8093, Switzerland. E-mail: kwuertz@ethz.ch

[#] K. Wuertz-Kozak and O. N. Hausmann contributed equally

Internal control screening

Method: To identify the most stable reference gene in human non-degenerated and degenerated IVD tissue, geNorm[1] software was used. Nine reference genes were investigated: ACTB, GAPDH, GUSB, RPL4, RPL13A, SDHA, TBP, YWHAZ and 18s (Table S2) in human non-degenerated (2xAF, 2xNP) and degenerated (1xAF Pfirrmann Grade 4, 1xNP Pfirrmann Grade 4, 1xAF Pfirrmann Grade 5, 1xNP Pfirrmann Grade 5) IVD tissue using standard qPCR procedure. GUSB was not detected in one of the analyzed samples and was thus excluded from the geNorm analysis.

Results: geNorm identified YWHAZ, GAPDH and PRL4 as the most stable reference genes (Fig. S1) in the analyzed sample set. In contrast, the commonly used reference genes ACTB, and TBP showed more variation in the expression between the samples (Fig. S1).

Conclusion: Based on these results and due to its availability and compatibility with other studies currently being conducted in our laboratory (data not showed), YWHAZ was selected as the reference gene for this study.

TRP channel expression: tissue versus isolated cells

Method: To evaluate whether the mRNA expression of TRP channels is affected by enzymatic digestion for cell isolation, five out of 22 degenerated IVD samples with sufficient size were not only used for direct RNA isolation (as described in the main document material and methods section), but also for IVD cell isolation. After intra-operative excision, IVD sample were transported to the laboratory either in RNAlater (Thermo Fisher, Switzerland) or in DMEM/F12 (Sigma-Aldrich, Switzerland) with 3% antibiotic-antimycotic (Anti-Anti) solution (300 U/ml Penicillin; 300 µg/ml Streptomycin; 3.75 µg/ml Amphotericin B; Gibco, USA). Fresh samples were washed in sterile phosphate-buffered saline (PBS) solution and cut into fine pieces before incubation in an enzymatic digestion solution (0.3% Collagenase NB4 (Serva, Germany), 0.2% Dispase II (Roche Diagnostics, Germany), 3% Anti-Anti in PBS) for 12h at 37°C, 5% CO_2, 21% O_2. Following filtration of the tissue digest through cell strainers (70 and 100 µm), the cells were pelleted (1000 rpm, 10 minutes, 20°C with a PBS washing step in-between) and lysed in 1 mL of TRIzol (Thermo Fisher, Switzerland). RNA was extracted by TRIzol/chloroform method, followed by affinity-based purification. Cell pellets were supplemented with 200 µL chloroform (1-part chloroform to 5 parts sample) as described above. One part of isopropanol was added (1:1 ratio) to the aqueous phase and samples were shaken 10 times, incubated for 5 min at RT and centrifuged (4°C, 12000g, 10 min). Next, the supernatant was removed and the pellet was mixed with 70% Ethanol, vortexed and centrifuged again (4°C, 12000g, 5 min). Lastly, the Ethanol was removed and samples were left for 5 min at the RT to allow the remaining Ethanol to evaporate. Finally, extracted RNA was re-suspended in 25 µL of RNAse-free water.

Results: No statistically significant differences ($p>0.05$, Table S3) were found in the TRP channels mRNA expression between IVD tissue and cells collected directly after isolation (Fig. S2).

Conclusion: We were able to show that the enzymatic digestion of the IVD tissue did not affect the mRNA expression of the selected TRP channels (and PDK1, Fig. S2). This finding may be important when considering future experimental set-ups, as well as accessibility to certain laboratory equipment and consumables, including liquid nitrogen containers in hospitals.

Transient Receptor Potential (TRP) polycystin (P) channel subfamily

Since it was reviewed that little evidence exist for the 11-TM subfamily (PKD1) to be forming functional ion channels, PKD1 was excluded from the TRP channel family, leading to a change

in nomenclature[2]. However, it was recently proposed that PDK1 may be necessary for the PDK2 in order to produce cation currents[3], hence PKD1 should not be excluded from the analysis when evaluating the TRPP1 (PDK2) channel. Consequently, old and new nomenclature can still be find in publications, as well as in biotechnology products. Hence, caution must be taken when reviewing the literature or purchasing biological assays. To avoid the confusion, the old and new nomenclature is provided in the Table S4.

Tables and Figures

Table S1 Results of the TaqMan array test of 29 TRP channel targets, displayed as Ct values

**Gene**	**Donor 1<br><br>AF PDG 4**	**Donor 2<br><br>NP PDG 4**	**Donor 3<br><br>AF PDG 5**	**Donor 4<br><br>NP PDG 5**	**Donor 5<br><br>AF N-DEG**	**Donor 6<br><br>NP N-DEG**	**Donor 7<br><br>AF N-DEG**	**Donor 8<br><br>NP N-DEG**	**Selection**
TRPA1	n.d	25.93	28.16	28.42	n.d	n.d	n.d	n.d	
TRPC1	18.84	18.05	17.46	20.61	23.30	24.49	29.20	26.24	
TRPC2	26.87	22.14	24.96	28.68	26.96	28.64	31.38	26.31	
TRPC3	26.75	19.67	21.06	23.06	n.d	n.d	n.d	n.d	
TRPC4	30.05	23.85	24.45	26.20	n.d	n.d	n.d	n.d	
TRPC5	n.d	n.d	n.d	n.d	n.d	n.d	n.d	n.d	
TRPC6	27.14	19.95	19.91	23.24	30.71	n.d	n.d	n.d	
TRPC7	28.98	n.d	30.00	n.d	n.d	n.d	n.d	n.d	
TRPM1	n.d	29.40	n.d	30.06	n.d	n.d	n.d	n.d	
TRPM2	26.07	20.91	22.83	24.45	n.d	n.d	n.d	n.d	
TRPM3	n.d	20.98	24.69	25.80	n.d	n.d	n.d	n.d	
TRPM4	22.58	19.38	21.05	22.68	n.d	27.85	n.d	n.d	
TRPM5	n.d	n.d	n.d	n.d	n.d	n.d	n.d	n.d	
TRPM6	25.61	22.26	21.44	26.36	n.d	33.19	n.d	n.d	
TRPM7	18.62	16.35	17.51	19.96	23.90	24.21	n.d	27.42	
TRPM8	30.64	26.95	n.d	n.d	n.d	n.d	n.d	n.d	
TRPML1	21.72	18.54	19.91	21.90	27.50	27.87	n.d	29.71	
TRPML2	22.08	21.96	23.27	22.80	27.10	28.43	n.d	n.d	
TRPML3	21.30	20.20	20.93	21.83	27.70	27.87	n.d	28.93	
PDK1	19.10	18.66	19.00	19.14	26.55	26.24	29.70	28.96	
TRPP1 (PKD2)	18.42	16.50	16.89	19.11	24.48	24.44	27.95	27.38	
TRPP2 (PKD2L1)	30.57	29.22	n.d	n.d	n.d	n.d	n.d	n.d	
TRPP3 (PKD2L2)	30.08	n.d	n.d	n.d	n.d	n.d	n.d	n.d	
TRPV1	22.33	20.94	21.69	23.52	27.41	29.05	31.10	30.78	
TRPV2	24.38	19.58	21.58	23.53	n.d	n.d	n.d	n.d	

TRPV3	25.81	25.03	25.91	27.01	28.96	31.33	n.d	30.64	
TRPV4	16.19	17.57	18.20	17.52	23.25	24.14	28.60	26.07	
TRPV5	29.59	26.55	28.28	28.22	n.d	30.55	n.d	n.d	
TRPV6	41.88	27.78	n.d	n.d	n.d	n.d	n.d	n.d	

AF = annulus fibrosus; NP = nucleus pulposus; PDG = Pfirrmann degeneration grade; N-DEG = non-degenerated; n.d = non-detectable; TRP = transient receptor potential channel; filled field in the *Selection* column indicates that the target was selected for further tests

Table S2 TaqMan primers used for the reference gene screening using qPCR analysis

**Gene**	**Gene class**	**Primer number**
ACTB	Internal control	Hs01060665_g1
GAPDH	Internal control	Hs02758991_g1
GUSB	Internal control	Hs00939627_m1
RPL4	Internal control	Hs03044646_g1
RPL13A	Internal control	Hs04194366_g1
SDHA	Internal control	Hs00188166_m1
TBP	Internal control	Hs00427620_m1
YWHAZ	Internal control	Hs01122445_g1
18s	Internal control	Hs99999901_s1

ACTB = Beta Actin; GAPDH = Glyceraldehyde-3-phosphate Dehydrogenase; GUSB = Glucuronidase Beta; RPL4 = Ribosomal Protein L4; RPL13A = Ribosomal Protein L13a; SDHA = Succinate Dehydrogenase Complex Flavoprotein Subunit A; TBP = TATA-Box Binding Protein; YWHAZ = Tyrosine 3-Monooxygenase/Tryptophan 5-Monooxygenase Activation Protein Zeta

Table S3 The statistical significance values (p-values) for the tested TRP channels comparing dCt values of the mRNA expression between the values obtained from tissue versus cells samples, calculated with Monte Carlo Randomization test with a significance level at $p<0.05$

**Target**	**p-value**
TRPC1	0.13
TRPC3	0.25
TRPC4	1
TRPC6	0.25
TRPM2	0.75
TRPM7	1
TRPML1	0.24
TRPML2	0.88
TRPP1	0.07
TRPP2	0.56
TRPV1	0.06
TRPV4	0.18

Table S4 TRPP channel subfamily nomenclature

Gene alias	Former name	Current name
PKD1	TRPP1	PKD1
PKD2	TRPP2	TRPP1
PKD2L1	TRPP3	TRPP2
PKD2L2	TRPP5	TRPP3

PKD1 = Polycystins include putative 11-TM also called the PC1 family; PKD2 = polycystic kidney disease 2 also called polycystin 2 (PC2); PKD2L1 = Polycystic kidney disease 2-like 1; PKD2L1 = Polycystic kidney disease 2-like 2; TRPP (1,2,3,5) = Transient receptor potential channel subfamily P member 1, 2, 3, 5

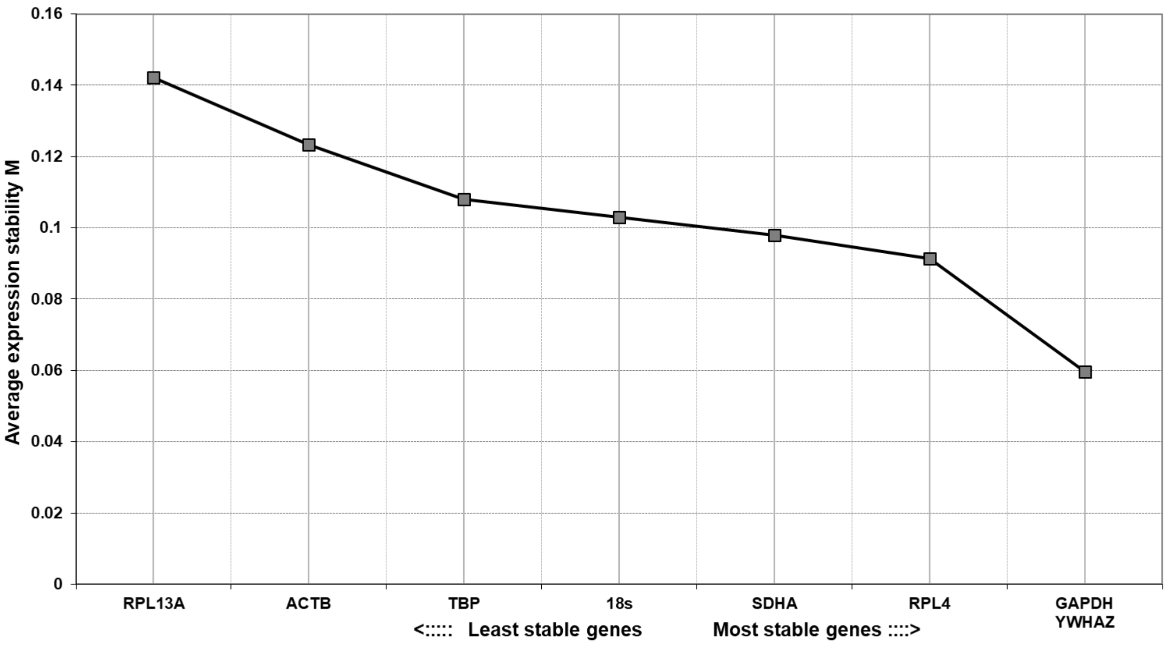

Fig. S1 The average expression stability values (M) obtained using geNorm tool. The expression of ACTB, GAPDH, RPL4, RPL13A, SDHA, TBP, YWHAZ and 18s was tested on human non-degenerated (2x AF, 2xNP) and degenerated (2xAF, 2xNP) IVD tissue. The most stable genes are presented on the right and the least stable on the left.

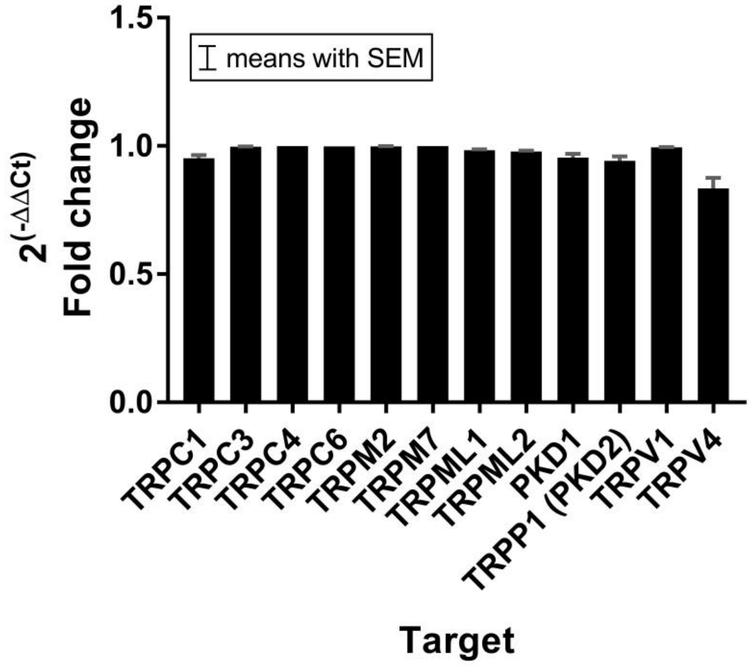

Fig. S2 Comparison of the mRNA expression of selected TRP channels in the human degenerated IVD samples between the RNA samples obtained directly from the tissue (via pulverization in the liquid nitrogen) and non-passaged cells (via enzymatic digestion). Results are presented as $2^{-\Delta\Delta Ct}$ values of mRNA normalized to the tissue samples

References

1 Vandesompele, J. *et al.* Accurate normalization of real-time quantitative RT-PCR data by geometric averaging of multiple internal control genes. *Genome Biol* **3**, RESEARCH0034 (2002).

2 Wu, L. J., Sweet, T. B. & Clapham, D. E. International Union of Basic and Clinical Pharmacology. LXXVI. Current progress in the mammalian TRP ion channel family. *Pharmacol Rev* **62**, 381-404, doi:10.1124/pr.110.002725 (2010).

3 Hanaoka, K. *et al.* Co-assembly of polycystin-1 and -2 produces unique cation-permeable currents. *Nature* **408**, 990-994, doi:10.1038/35050128 (2000).

Acknowledgments

The study was financially supported by the Swiss National Science Foundation (SNF PP00P2_163678/1) as well as the Spine Society of Europe (Eurospine 2016_4).

Non-degenerated tissue was provided by the McGill Scoliosis and Spine Group.

Degenerated tissue was provided by the Hirslanden Klinik St. Anna.

Asymptomatic degenerated and non-degenerated entire disc sections used for immunohistological analysis were provided by Andreas Nerlich from the Institute for Pathology at the Klinikum Bogenhausen Munich.

Authors Contributions

A.S. performed experiments, collected data, interpreted the results, prepared figures and tables, and wrote the manuscript. W.H. did the statistical analysis and assisted in results interpretation. A.K. developed the IHC protocols and performed the immunohistochemistry and data analysis. P.J. and O.N.H managed the collection of degenerated samples and provided clinical relevance. L.H. managed the collection of non-degenerated samples. H.C. assisted in non-degenerated sample collection and sample processing. K.W.K. designed the study, wrote the manuscript and secured funding. All authors reviewed, edited and approved the final manuscript.

Competing Interests

The authors declare that they have no competing financial and non-financial interests.

Ethical approval

All procedures performed in studies involving human participants were in accordance with the ethical standards of the institutional and/or national research committee and with the 1964 Helsinki Declaration and its later amendments or comparable ethical standards.
